# Supplementary material for: Evaluation of the impact of chemical control on the ecology of Rattus norvegicus of an urban community in Salvador, Brazil
Source: PLoS One. 2022 Jul 20;17(7):e0270568. doi: 10.1371/journal.pone.0270568 (PMC9299319; doi:10.1371/journal.pone.0270568)

**1 – Supplementary material**

- 1. **– Supplementary methods: Zero-inflated beta-regression model to evaluate association between chemical intervention variables and capture success**

Among the various probability distributions of zero-inflated beta regression is the possibility to adjust data ranging from zero to one as rates or proportions. However, these models are only useful for inference values ​​between an open range of zero and one, that is, ranges that do not include zero and one. Smithson and Verkuilen (59) proposed a transformation of these values ​​to apply a simple beta regression, but this solution does not solve the problem of data sets with many zeros and one only transforms the problem. Due to the characteristic of success in catching many absences (~ 50%), we used a zero inflated beta packet in R using zoib (36). The package uses Bayesian inference to estimate the effects of the explanatory variables on the response variable and on the values ​​of zero, so it was also possible to test if there is any relation with the increase in the number of baits and the number of absences found. The performance of the model was checked by the convergence of the Markov chains using the traceplot function and by the autocorrelation plot (autocorr.plot). For more information on checking the model check (36). The identity of the point was considered as a random effect, since in each capture campaign they were sampled. The Odds Ratio of each parameter was calculated, and confidence intervals that included 1 were considered non-significant.

|  | Β | 2.50% | 97.50% |
| --- | --- | --- | --- |
| **Intercept** | **0.305088** | **0.178491** | **0.541624** |
| Amount of bait | 1.02713 | 0.984525 | 1.069847 |
| **1st application of contact powder** | **2.123828** | **1.242872** | **3.550674** |
| 2nd application of contact powder | 1.206034 | 0.564745 | 2.29707 |
| 3rd application of contact powder | 0.787333 | 0.269418 | 2.042825 |
| Campaign 2 (3 months post-intervention) | 1.092564 | 0.797779 | 1.635151 |
| Campaign 3 (6 months post-intervention) | 0.817365 | 0.452297 | 1.413422 |
| Precipitation | 1.000782 | 0.981665 | 1.02161 |
| Valley 2 | 1.228836 | 0.610363 | 2.259821 |
| Valley 3 | 0.795309 | 0.514245 | 1.272639 |
| Intercept | 1.478955 | 0.325851 | 6.93095 |
| Amount of bait | 0.920717 | 0.810475 | 1.039757 |
| **1st application of contact powder** | 0.461334 | 0.142897 | 1.295325 |
| 2nd application of contact powder | 0.32781 | 0.046948 | 1.749861 |
| 3rd application of contact powder | 0.225104 | 0.003057 | 4.693077 |
| Campaign 2 (3 months post-intervention) | 0.94017 | 0.394905 | 2.173765 |
| Campaign 3 (6 months post-intervention) | 0.811922 | 0.248982 | 3.473337 |
| Precipitation | 1.00179 | 0.95351 | 1.046527 |
| Valley 2 | 0.590805 | 0.102466 | 2.689212 |
| Valley 3 | 2.299341 | 0.880098 | 6.964962 |
| **D** | **11.76534** | **7.905605** | **17.25162** |
| **Sigma** | **1.065785** | **1.00004** | **1.289708** |

1.1.2 – autocorrelation graph


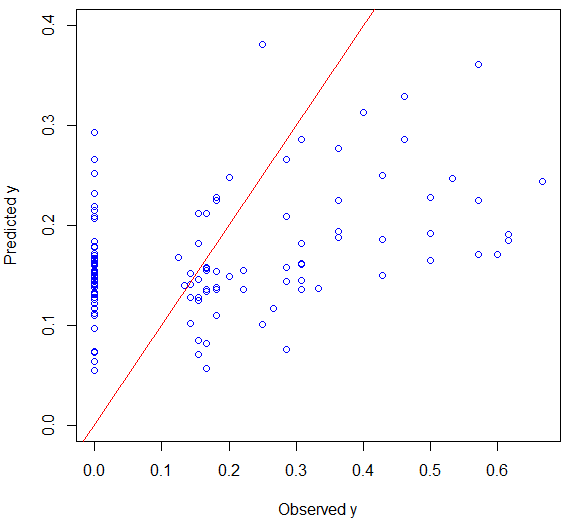

Supplement: S1 File — (DOCX) [file pone.0270568.s001.docx]
